# Total Neoadjuvant Therapy for Rectal Cancer in the CAO/ARO/AIO-12 Randomized Phase 2 Trial: Early Surrogate Endpoints Revisited

**DOI:** 10.3390/cancers14153658

**Published:** 2022-07-27

**Authors:** Markus Diefenhardt, Anke Schlenska-Lange, Thomas Kuhnt, Simon Kirste, Pompiliu Piso, Wolf O. Bechstein, Guido Hildebrandt, Michael Ghadimi, Ralf-Dieter Hofheinz, Claus Rödel, Emmanouil Fokas

**Affiliations:** 1 Department of Radiotherapy and Oncology, University of Frankfurt, 60596 Frankfurt, Germany; clausmichael.roedel@kgu.de (C.R.); emmanouil.fokas@kgu.de (E.F.); 2 Frankfurt Cancer Institute, 60596 Frankfurt, Germany; 3 Department of Haematology and Oncology, Barmherzige Brüder Hospital Regensburg, 93049 Regensburg, Germany; anke.schlenska-lange@barmherzige-regensburg.de; 4 Department of Radiation Oncology, University Hospital Leipzig, 04103 Leipzig, Germany; thomas.kuhnt@medizin.uni-leipzig.de; 5 Department of Radiation Oncology, Faculty of Medicine, Medical Center University of Freiburg, 79098 Freiburg, Germany; simon.kirste@uniklinik-freiburg.de; 6 German Cancer Research Center (DKFZ), German Cancer Consortium (DKTK), Partner Site: Freiburg, 69120 Heidelberg, Germany; 7 Department of General and Visceral Surgery, Barmherzige Brüder Hospital, 93049 Regensburg, Germany; allgemeinchirurgie@barmherzige-regensburg.de; 8 Department of General and Visceral Surgery, University of Frankfurt, 60596 Frankfurt, Germany; wolf.bechstein@kgu.de; 9 Department of Radiotherapy, University of Rostock, 18051 Rostock, Germany; guido.hildebrandt@med.uni-rostock.de; 10 Department of General and Visceral Surgery, University Medical Center Göttingen, 37075 Göttingen, Germany; chirurgie.sekretariat@med.uni-goettingen.de; 11 Department of Medical Oncology, University Hospital Mannheim, 68167 Mannheim, Germany; ralf-dieter.hofheinz@medma.uni-heidelberg.de; 12 German Cancer Research Center (DKFZ), German Cancer Consortium (DKTK), Partner Site: Frankfurt, 69120 Heidelberg, Germany

**Keywords:** locally advanced rectal cancer, clinical trial, early surrogate endpoints, tumor regression, disease-free survival

## Abstract

**Simple Summary:**

Multimodal treatment of rectal cancer is undergoing dynamic change. In phase II/III multimodal rectal cancer trials, long-term survival remains the most objective endpoint for reporting treatment efficacy, but long follow-up is required, and there is a risk that the study results will lose scientific significance over time. To address these limitations, early surrogate endpoints are increasingly used to identify treatment efficacy at an earlier timepoint. We here report the prognostic role of pCR (pathological complete response), TRG (tumor regression grade) and NAR score (neoadjuvant rectal score) for DFS (disease-free survival) in the CAO/ARO/AIO-12 trial. Surrogate markers were significant prognostic factors for DFS, but the higher pCR rate und improved TRG in trial Arm B did not lead to improved survival compared to Arm A. Therefore, early surrogate marker correlated with clinical outcome in the CAO/ARO/AIO-12 trial, but the early differences in pCR and TRG did not translate into a survival benefit.

**Abstract:**

Background: Early efficacy outcome measures in rectal cancer after total neoadjuvant treatment are increasingly investigated. We examined the prognostic role of pathological complete response (pCR), tumor regression grading (TRG) and neoadjuvant rectal (NAR) score for disease-free survival (DFS) in patients with rectal carcinoma treated within the CAO/ARO/AIO-12 randomized phase 2 trial. Methods: Distribution of pCR, TRG and NAR score was analyzed using the Pearson’s chi-squared test. Univariable analyses were performed using the log-rank test, stratified by treatment arm. Discrimination ability of non-pCR for DFS was assessed by analyzing the ROC curve as a function of time. Results: Of the 311 patients enrolled, 306 patients were evaluable (Arm A:156, Arm B:150). After a median follow-up of 43 months, the 3-year DFS was 73% in both groups (HR, 0.95, 95% CI, 0.63–1.45, *p* = 0.82). pCR tended to be higher in Arm B (17% vs. 25%, *p* = 0.086). In both treatment arms, pCR, TRG and NAR were significant prognostic factors for DFS, whereas survival in subgroups defined by pCR, TRG or NAR did not significantly differ between the treatment arms. The discrimination ability of non-pCR for DFS remained constant over time (C-Index 0.58) but was slightly better in Arm B (0.61 vs. 0.56). Conclusion: Although pCR, TRG and NAR were strong prognostic factors for DFS in the CAO/ARO/AIO-12 trial, their value in selecting one TNT approach over another could not be confirmed. Hence, the conclusion of a long-term survival benefit of one treatment arm based on early surrogate endpoints should be stated with caution.

## 1. Introduction

Neoadjuvant chemoradiotherapy (CRT), or short-course RT (SCRT), improved local control in locally advanced rectal cancer, but the benefit on long-term disease-free (DFS) and overall survival (OS) was limited [1,2,3,4]. The value of additional adjuvant chemotherapy (CT) after neoadjuvant therapy and total mesorectal excision (TME) surgery remains unclear for DFS and OS, and the addition of oxaliplatin to neoadjuvant CRT provided inconsistent results [5,6,7,8,9]. More recently, total neoadjuvant treatment (TNT), e.g., SCRT/CRT with either induction or consolidation CT, showed improved distant metastasis-free and disease-free survival (DFS) [10,11,12,13]. Furthermore, compared to classical SCRT/CRT, TNT can enhance clinical and pathological complete response (c/pCR) rates to explore organ preservation [14].

In principle, OS is the most objective endpoint in phase 3 cancer trials, but large cohorts of patients and costly, extensive, and long follow-up protocols are needed to provide accurate long-term survival data [4]. Furthermore, survival analysis can be confounded by successful treatment of disease recurrences or non-cancer related death, particularly in elderly patients. To overcome these limitations and identify promising therapeutic approaches at an earlier stage, several early or intermediate efficacy endpoints have been proposed [4,15].

Pathological complete remission (pCR), neoadjuvant rectal (NAR) score, and tumor regression grading (TRG) have been established as early surrogate endpoints after standard neoadjuvant CRT to reflect both tumor biology and treatment efficacy as reported in several randomized phase II–III trials [4,16,17]. However, the value of these early surrogate endpoints in the era of TNT, with varying sequences and intervals between treatment components, remains largely unexplored. Here, we investigated the prognostic value of these early endpoints in the CAO/ARO/AIO-12 randomized phase 2 trial. In this study, TNT with upfront CRT followed by consolidation CT resulted in higher pCR (primary endpoint) compared to induction CT followed by CRT but did not impact on secondary endpoints such as DFS and OS [18].

## 2. Materials and Methods

### 2.1. Patient Selection

The CAO/ARO/AIO-12 was a multicenter, randomized, phase 2 trial (ClinicalTrials.gov, accessed on 7 June 2022, NCT02363374). Inclusion criteria included patients ≥18 years old with rectal adenocarcinoma, ECOG performance status 0–1, cT3 tumors < 6cm from the anal verge, cT3 tumors in the middle third of the rectum (≥6–12 cm at rigid rectoscopy) with extramural tumor spread into the mesorectal fat of more than 5 mm (>cT3b), cT4 tumors, or clinical lymph node involvement. Evaluation of clinical nodal involvement was based on mandatory magnetic resonance imaging. Distant metastases were excluded by CT scan of the abdomen and chest. Laboratory tests for adequate organ function were conducted prior to enrollment in the trial.

The CAO/ARO/AIO-12 trial was approved by the Ethics’ Committee of the University Hospital Frankfurt on 19 January 2015 (Ethic Code: 406/14, EudraCT-Nr: 2011-006310-13), and all patients signed a consent.

### 2.2. Treatment

Patients were randomly assigned to treatment Arm A for induction CT prior to CRT or to Arm B for CRT followed by consolidation CT. Radiotherapy was prescribed to the primary tumor and the mesorectal, presacral, and internal iliac lymph nodes to a total dose of 50,4 Gy in 28 fractions. Fluorouracil as continuous infusion (250 mg/m^2^) on day 1–14 and day 22–35 intensified with oxaliplatin (50 mg/m^2^) on day 1, 8, 22 and 29 were administered simultaneously during radiotherapy. Oxaliplatin 100 mg/m^2^ as a two-hour infusion, followed by a 2 h infusion of leucovorin (400 mg/m^2^) and a continuous 46 h infusion of fluorouracil (2400 mg/m^2^), repeated on day 15 for a total of three cycles was administered as induction or consolidation CT. If necessary, due to toxicity, doses were modified according to the trial protocol. Independently of primary tumor response, on day 123 after initiation of TNT, total mesorectal excision (TME) surgery was scheduled. Nonoperative management was considered a protocol violation, but 10 patients with clinical complete response after TNT rejected surgery. Adjuvant CT after curative surgery was not recommended. 

### 2.3. Early Efficacy Endpoints

The analysis of the primary endpoint, pathological complete response (pCR, ypT0N0) has already been reported [19]. TRG was recorded prospectively in both arms of the study according to Dworak et al. [20]. The neoadjuvant rectal score (NAR) incorporates cT to account for tumor downstaging, and ypT and ypN that are influenced directly by preoperative treatment [15]. The NAR formula is as follows: NAR = (5 pN − 3*(cT-pT) + 12)/9.61, where cT in (1, 2, 3, 4), pT in (0, 1, 2, 3, 4) and pN in (0, 1, 2). NAR consists of 24 distinct scores that range from 0 to 100. For ypT-category and ypN-category, a relative weight of 3 and 5 was suggested to reflect the impact of these variables, based on the nomogram of Valentini [21]. The constant 12 is included to maintain all scores inside the brackets as positive. The scaling factor 9.61 was introduced to ensure that the final scores range from 0 to 100. The NAR score was classified as low (NAR < 8), intermediate (NAR = 8 − 16), and high (NAR > 16) as reported before [15].

### 2.4. Statistical Analysis

The distribution of pCR, TRG and NAR between both treatment arms was examined with the chi-squared test. The secondary endpoint, DFS, was defined as the time between randomization and the first of the following events: macroscopically incomplete surgery (R2 resection), locoregional or metastatic recurrence or death from any course.

We used the log-rank test to determine the prognostic role of pCR, NAR and TRG for DFS. DFS in subgroups defined by pCR, non-pCR and TRG 0/1, TRG 2/3, TRG 4, respectively, NAR low, intermediate, and high risk were compared with the log-rank test as well. Unadjusted subgroup analyses to identify potential different treatment effects in pCR and non-pCR subgroups were performed using the “subtee” package in R [22]. The methodology of time-dependent ROC curve analysis is described in the Supplementary Methods. Patients with missing values were excluded. Statistical analyses were performed with the SPSS 25 software (SPSS Inc., Chicago, IL, USA) and the R system, version 4.1 (packages: “subtee”, “risksetROC” and “timeroc”).

## 3. Results

### 3.1. Accrual and Patient Characteristics

From 15 June 2015 to 31 January 2018, 311 patients from 18 centers in Germany were recruited. Five patients proved ineligible after enrollment. Of the remaining 306 eligible patients, 156 patients were randomized to Group A (sequence CT/CRT/Surgery) and 150 patients to Group B (sequence CRT/CT/Surgery). All 156 patients started induction CT in Arm A, whereas consolidation CT in Arm B was started in 140 (93%) patients. In Arm A 151 (97%) patients proceeded to CRT and in Arm B 159 (99%) received CRT. 143 (92%) patients in Arm A and 143 (95%) patients in Arm B underwent surgery [18,19]. 

There were no significant differences in the distribution of any of the three parameters between the two TNT groups, albeit a trend toward higher pCR rates (*p* = 0.086) as well as high rates of TRG4 (27% vs. 19%) and low NAR score (36% vs. 26%) was observed in group B (Table 1).

### 3.2. Treatment Efficacy

The pCR rate, pathological evaluation, treatment toxicity, surgical morbidity, adherence to treatment as well as oncological outcomes have previously been reported [18,19].

After a median follow-up of 43 months (IQR, 35–49, range 35–60), 3-year DFS was 73% in both treatment arms; 73% (95% CI, 66–80) in Arm A vs. 73% (95% CI, 66–80) in Arm B, HR, 0.95, (95% CI, 0.63-1.45, *p* = 0.82).

**Figure 1:**
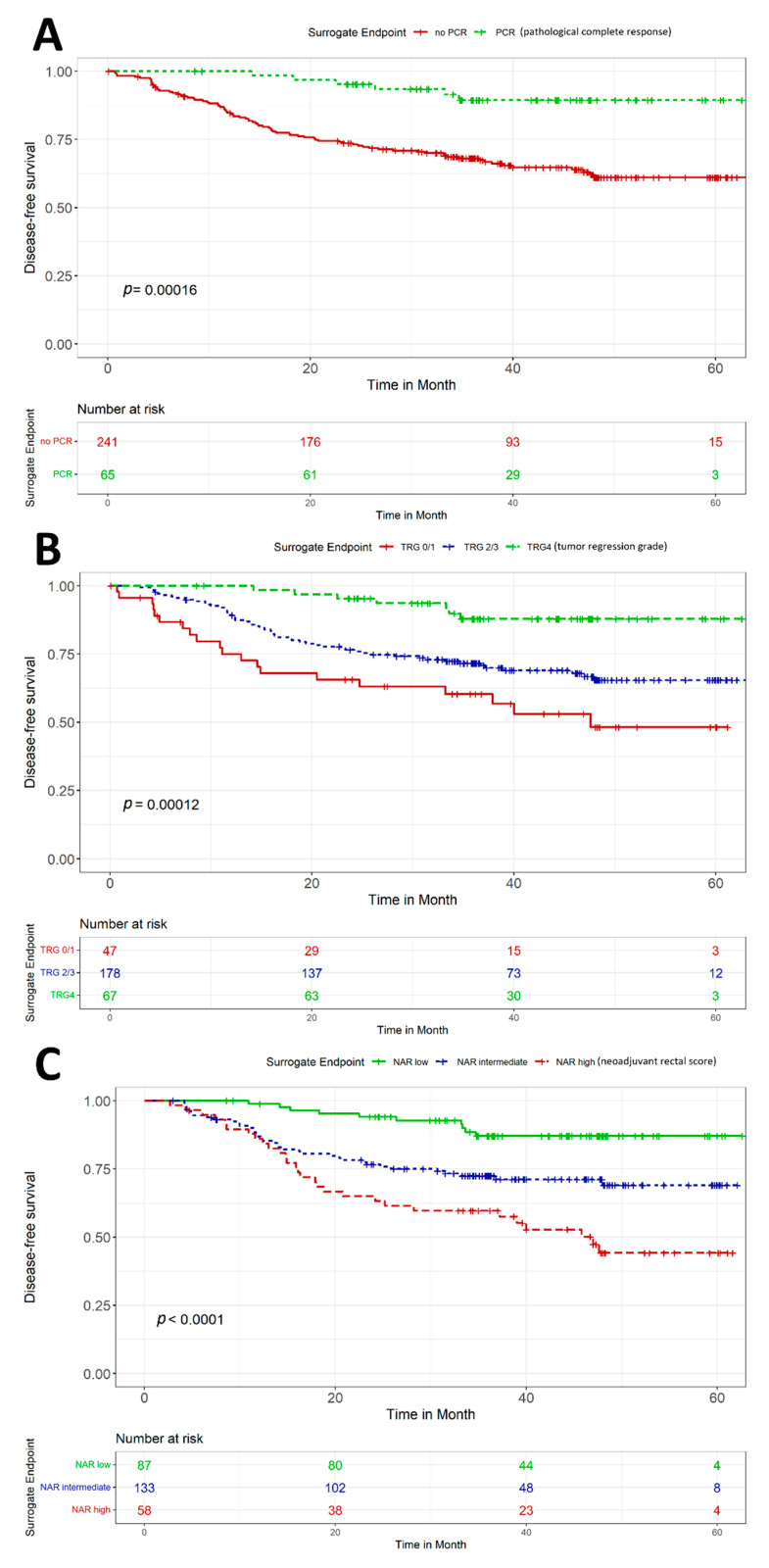
Prognostic significance of pCR (**A**), TRG (**B**) and NAR score (**C**) for disease-free survival. The log-rank test was used to assess statistical significance. The statistical test was two-sided.

Regarding the prognostic value of early endpoints, in univariate analysis, pCR, TRG, and NAR score were significantly associated with 3-year DFS in the entire cohort (Figure 1). The strong prognostic value of the early endpoints for DFS remained in both treatment groups (Table 2). Furthermore, we examined the prognostic impact of each of the subgroups of pCR, TRG and NAR score on the 3-year DFS separately, as shown in Table 3. We did not observe a significant difference between the two TNT regimens in terms of 3-year DFS for any of the subgroups of pCR, TRG, and NAR score.

**Figure 2:**
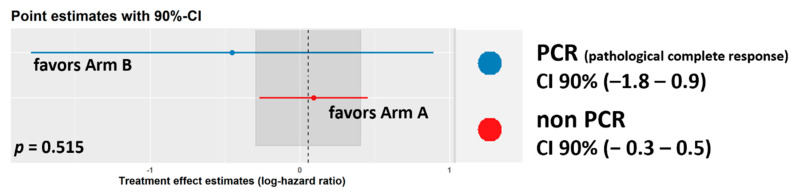
Plot of unadjusted treatment effects for pCR and non-pCR subgroups for disease-free survival. The 90% confidence interval is plotted. The overall treatment effect under the model with no treatment-subgroup interactions are plotted with a dashed line, as well as its confidence intervals plotted as a gray shaded area. Statistical significance was examined and plotted with the “subtee” package in R.

Further, using the unadjusted estimates for subgroups, we failed to detect a differential treatment effect when testing pCR vs. non-pCR (Figure 2). A discrimination ability test was performed as shown in the Supplementary Materials and in Figure S1.

## 4. Discussion

In this post hoc, secondary analysis of the CAO/ARO/AIO-12 trial, we examined the prognostic impact of the early efficacy measures pCR, TRG and NAR score for DFS. Albeit early outcome measures were significantly prognostic for 3-year DFS in the entire cohort, with each TNT arm separately, there were no significant differences in early efficacy endpoints and 3-year DFS between the two TNT groups, or for any of the subgroups for pCR, TRG and NAR score [18,19]. In the initial report of the primary endpoint of the CAO/ARO/AIO-12, we found significantly higher pCR compared to an assumed historical pCR rate of 15% after standard preoperative fluorouracil-based CRT (*p* < 0.001) in Arm B but not in Arm A, based on the modified “pick-the-winner” statistical trial design. Notably, improved pCR did not translate to better oncologic outcome after a median follow-up of 43 months (DFS was 73% in both TNT) [19].

Previous clinical studies have reported heterogeneous results regardless of whether pCR could serve as a surrogate for DFS/OS. Historically, the POLISH I trial and the TROG 01.04 trial compared SCRT followed by surgery within one week and adjuvant CT versus long-course CRT followed by delayed surgery and adjuvant CT. Both trials reported significantly higher pCR rates after CRT and delayed surgery with no significant differences in DFS and OS [23,24]. More recently, the STOCKHOLM III trial investigated delayed versus immediate surgery after SCRT and reported improved pCR rates but no DFS/OS benefit [25,26,27] (Table 4). These data reflect the limitation of pCR as a surrogate endpoint for DFS/OS [28], which was also shown in the meta-analysis of 22 studies in 10,050 patients by Petrelli et al. [29].

With respect to intensified neoadjuvant CRT regimen, our previous randomized CAO/ARO/AIO-04 trial showed that pCR was achieved in 17% of patients treated with oxaliplatin/fluorouracil-based CRT vs. 13% (*p* = 0.038) treated within fluorouracil-based CRT. This higher pCR rate correlated with superior 3-year DFS of the experimental arm [5,30] (Table 4). The FORWARC trial also reported improved pCR rates by the addition of oxaliplatin to neoadjuvant fluorouracil-based CRT (28% vs. 14%), however without a significant improvement in 3-year DFS [31,32]. Conversely, a Chinese trial by Jia et al. reported lower incidence of distant metastasis but no increase in pCR rates through intensified neoadjuvant CRT with oxaliplatin [33].

Regarding the value of pCR as a surrogate measure for survival in TNT, the recently reported clinical trials also provided heterogenous results. In the RAPIDO trial, higher pCR rates translated to improved DFS and lower incidence of distant metastasis [10]. In the PRODIGE-23 trial, pCR, TRG 4 and low-risk NAR score in the TNT arm correlated with improved DFS, whereas in the STELLAR trial, improved pCR was not associated with better DFS [12,13] (Table 4).

Thus, tumor response as a dynamic process is not only affected by tumor- and patient-related factors, such as tumor size, molecular profile, histology, or host’s immune system. Treatment-related factors, RT dose and fractionation, administration of concurrent CT and/or use of induction/consolidation CT and, most importantly, the time interval between radiotherapy and response assessment are critical to tumor response. Tumor response, as measured by pCR, may predict favorable long-term survival for individual patients within a certain treatment protocol, but implication for superior outcome in comparative trials cannot be necessarily concluded therefrom [4].

Accordingly, in our trial, the difference in pCR between the two TNT groups likely reflects the different interval and continuously ongoing response from the last radiotherapy fraction to surgery, which was (median) 85 days in Arm B vs. 42 days in Arm A. In addition, reduced adherence to CRT following induction CT, as well as selection and expansion of more radiation-resistant clones by induction CT (which may alter apoptotic pathways, upregulate epidermal growth factor receptor expression, and affect angiogenesis and stromal proliferation) may have contributed to less pCR in Arm A [34].

Regarding non-pCR, the discrimination ability for DFS remained largely constant over the follow-up period but differed slightly between the two treatment arms. The discrimination ability seems to be lower in Arm A than in Arm B (AUC 55 vs. 61), and DFS for non-pCR was higher compared to Arm B (69.5% vs. 66.3%), suggesting that the non-pCR subgroup in Arm A included good prognostic patients that would have developed pCR with a longer interval to response assessment [19].

Unlike pCR, TRG and the NAR score classifies tumor response more gradually beyond a simple binary system that may reflect treatment efficacy better and may have a greater ability than pCR to predict DFS or OS, as proposed by Yothers et al. [4,35]. Even if the surrogacy of TRG and NAR for improved DFS has been validated in the CAO/ARO/AIO-04 trial [16,17] and a significant trend to higher tumor regression and low NAR score correlated with improved DFS and lower incidence of distant metastasis in the PRODIGE-23 trial [12], potential surrogacy of both parameters has not been broadly reported in recent trials (Table 4) [10,13,14]. Furthermore, assessment and reporting of TRG is heterogeneous, and no universally approved standardization method is accepted [4,36]. The NAR score was proposed by the NRG Oncology as a surrogate endpoint for DFS and OS [15]. As no improvement in overall survival has been reported in any of the recent clinical trials in rectal cancer, except for in the Stellar trial, the ability to validate NAR as a surrogate for survival remains quite limited. Even if the NAR score incorporates pre- and post-neoadjuvant CRT tumor extent, further analyses of its surrogacy based on the recent published TNT trials are lacking.

Our study has limitations. First, this study constitutes a post hoc analysis. Second, analyses of changes of discrimination ability over time for surrogate measures have thus far not been performed in rectal cancer. These analyses are based on highly complex mathematical models, and heterogenous statistic methodologies have been published [37,38,39]. Therefore, interpretation of the potential difference in discrimination ability between both treatment arms should be interpreted with cautious even if the slightly weaker DFS in patients with pCR in Arm A support the thesis of a weaker discrimination ability of non-pCR for DFS in Arm A. Third, a central pathologic review for tumor regression was not conducted.

## 5. Conclusions

In summary, pCR, TRG and NAR score were prognostic parameters for DFS in the entire cohort as well as in both arms of the CAO/ARO/AIO-12 trial and likely reflect (and unmask) different tumor biology. However, their value in selecting one TNT approach over another could not be confirmed, as a better response in TNT group B did not translate to superior DFS or OS. Altering the sequence and intervals between components in multimodal rectal treatment may have substantial impact on early efficacy endpoints, but thus far, significant differences between the two TNT sequences on long-term clinical outcome measures after TME have not been reported. With the advent of TNT with selective nonoperative management (NOM) for patients with (near) clinical complete response, as reported in the recent OPRA trial [14] and currently investigated in our ongoing ACO/ARO/AIO-18.1 trial (NCT04246684), sustained local control without regrowth (i.e., TME-free survival) and disease-free survival including NOM and events of salvage surgery [4] have been incorporated as relevant clinical endpoints.

## Figures and Tables

**Table 1:** Distribution of pCR, TRG and NAR score in the two treatment arms of the CAO/ARO/AIO-12 trial.

Parameter	Total *n* = 306	TNT (Total Neoadjuvant Treatment)—Arm A (CT(Chemotherapy/CRT(Chemoradiotherapy) *n* (%)	TNT—Arm B (CRT/CT) *n* (%)	*p* Value *
pCR	65	27 (17)	38 (25)	
No pCR	241	129 (83)	112 (75)	0.086
TRG 4	67	28 (19)	39 (27)	
TRG 2 + 3	178	93 (63)	85 (59)	
TRG 0 + 1	47	26 (18)	21 (14)	0.261
NAR Low score	87	37 (26)	50 (36)	
NAR intermediate score	133	71 (51)	62 (45)	
NAR High score	58	32 (23)	26 (19)	0.206

Abbreviations: CRT, chemoradiotherapy; CT, chemotherapy; pCR, pathological complete response; TRG, tumor regression grading; NAR, neoadjuvant rectal; *n*, number * Distribution of the parameters according to the treatment arm was calculated using the chi-squared test.

**Table 2:** Distribution of pCR, TRG and NAR score in the two treatment arms of the CAO/ARO/AIO-12 trial.

Parameter	*n* (%) Arm A	3-Year DFS(Disease-Free Survival) * (% (95% CI (Confidence Interval))) Arm A (CT(Chemotherapy/CRT(Chemoradiotherapy)))	*p* Value	*n* (%) Arm B	3-Year DFS * (% (95% CI)) Arm B (CRT/CT)	*p* Value
pCR	27 (17)	86.5 (73.1–100)		38 (25)	91.2 (82.2–100)	
No pCR	129 (83)	69.5 (61.9–78.1)	**0.037**	112 (75)	66.3 (57.9–76.0)	**0.001**
TRG 4	28 (19)	87.1 (74.3–100)		39 (27)	88.5 (78.5–99.8)	
TRG 2 + 3	93 (63)	73.5 (65.0–83.2)		85 (59)	69.8 (60.5–80.5)	
TRG 0 + 1	26 (18)	58.1 (41.2–82.0)	**0.027**	21 (14)	63.7 (45.5–89.3)	**0.003**
NAR low score	37 (26)	84.3 (72.4–98.2)		50 (36)	89.0 (80.4–98.6)	
NAR intermediate score	71 (51)	76.0 (66.3–87.0)		62 (45)	68.5 (57.7–81.4)	
NAR high score	32 (23)	59.4 (44.6–79.1)	**0.002**	26 (19)	60.1 (43.7–82.7)	**0.001**

Abbreviations: DFS, disease-free survival; CRT, chemoradiotherapy; CT, chemotherapy; pCR, pathological complete response; TRG, tumor regression grading; NAR, neoadjuvant rectal; N, number. * The log-rank test was used to calculate statistical significance stratified by treatment arm. The statistical test was two-sided.

**Table 3:** Prognostic impact of each subgroup of pCR, TRG and NAR score on disease-free survival.

Parameter	3-Year DFS(Disease-Free Survival) * (% (95% CI)(confidence Interval)) in Each Arm Separately				
	*n* (%) Arm A	TNT(Total Neoadjuvant Treatment)—Arm A (CT(Chemotherapy/CRT(Chemoradiotherapy)))	*n* (%) Arm B	TNT—Arm B (CRT/CT)	*p* Value
pCR	27 (17)	86.5 (73.1–100)	38 (25)	91.2 (82.2–100)	0.532
No pCR	129 (83)	69.5 (61.9–78.1)	112 (75)	66.3 (57.9–76.0)	0.673
TRG 4	28 (19)	87.1 (74.3–100)	39 (27)	88.5 (78.5–99.8)	0.771
TRG 2 + 3	93 (63)	73.5 (65.0–83.2)	85 (59)	69.8 (60.5–80.5)	0.822
TRG 0 + 1	26 (18)	58.1 (41.2–82.0)	21 (14)	63.7 (45.5–89.3)	0.741
NAR low score	37 (26)	84.3 (72.4–98.2)	50 (36)	89.0 (80.4–98.6)	0.520
NAR intermediate score	71 (51)	76.0 (66.3–87.0)	62 (45)	68.5 (57.7–81.4)	0.297
NAR high score	32 (23)	59.4 (44.6–79.1)	26 (19)	60.1 (43.7–82.7)	0.815

Abbreviations: DFS, disease-free survival; CRT, chemoradiotherapy; CT, chemotherapy; pCR, pathological complete response; TRG, tumor regression grading; NAR, neoadjuvant rectal; N, number. * The log-rank test was used to calculate statistical significance stratified by treatment arm. The statistical test was two-sided.

**Table 4:** Association between reported surrogate endpoints (pCR, TRG and NAR score) and survival in major prospective rectal cancer trials.

Trials	Surrogate Endpoints					Survival			
	pCR (Pathological Complete Response) Rate		P	TRG (Tumor Regression Grade)	NAR (Neoadjuvent Rectal Score)	DFS (Disease-Free Survival)	LR (Local Recurrence)	DM (Distant Metastasis)	OS (Overall Survival)
	Arm A (%)	Arm B (%)							
**TNT (total neoadjuvant treatment)trials**									
GCR-3	13	14	n.s.	n.s.	n.r.	n.s.	n.s.	n.s.	n.s.
POLISH II	12	16	n.s.	n.r.	n.r.	n.s.	n.s.	n.s.	n.s.
RAPIDO	14	28	**<0.01**	n.r.	n.r.	**0.02**	n.s.	**<0.01**	n.s.
CAO/ARO/AIO-12	17	25	0.09	n.s.	n.s.	n.s.	n.s.	n.s.	n.s.
STELLAR	12	22	**<0.01**	n.r.	n.r.	n.s.	n.s.	n.s.	**0.03**
Prodige-23	12	28	**<0.01**	**<0.01**	**<0.01**	**0.03**	n.s.	**0.02**	n.s.
OPRA	41 ^#^	53 ^#^	**0.01**	n.r.	n.r.	n.s.	n.s.	n.s.	n.s.
Maréchal et al.	11	9	n.s.	n.s.	n.r.	n.r	n.r.	n.r.	n.r.
**Intensified** **neoadjuvant ** **CRT (chemoradiotherapy) trials**									
CAO/ARO/AIO-04	13	17	**0.04**	**<0.01**	**0.03**	**0.03**	n.s.	n.s.	n.s.
STAR-01	16	16	n.s.	n.r.	n.r.	n.s.	n.r.	n.r.	n.s.
NSABP R-04	18 °	21 °	n.s.	n.r.	n.r.	n.s.	n.s.	n.r.	n.s.
ACCORD-12	14	19	0.09	n.r.	n.r.	n.s.	n.s.	n.s.	n.s.
PETACC-06	12	14	n.s.	n.r.	n.r.	n.s.	n.s.	n.s.	n.s.
CHINESE	19	23	n.s.	n.r.	n.r.	n.s.	n.s.	**0.04**	n.s.
FORWARC	14 *	28 *	**<0.01**	n.r.	n.r.	n.s.	n.s.	n.s.	n.s.
**Interval to ** **Surgery trials**									
TIMING	18 ’’	38 ’’	**<0.01**	n.r.	n.r.	n.r.	n.r.	n.r.	n.r.
STOCKHOLM III	0.3 °°	10 °°	**<0.01**	**<0.01**	n.r.	n.s.	n.s.	n.s.	n.s.
**SCRT (short-course radiotherapy) vs. LCCRT (long-course radiotherapy)** **trials**									
POLISH I	1% ``	16% ``	**<0.01**	n.r.	n.r.	n.s.	n.s.	n.s.	n.s.
TROG 01.04	1% ``	15% ``	**<0.01**	n.r.	n.r.	n.r.	n.s.	n.s.	n.s.

* Fluorouracil radiotherapy arm vs. mFOLFOX6-radiotherapy arm; ^#^ TME-free survival; ° no OX arm vs. Ox arm; ’’ Group 1 vs. Group 4; ``Arm A—surgery within 7 days after 5 × 5 Gy vs. Arm B—surgery 4–6 weeks after chemoradiotherapy; °° SRT vs. SRT-delay. Abbreviations: pCR, pathological complete remission; TRG, tumor regression grade; NAR, neoadjuvant rectal score; DFS, disease-free survival; LR, incidence of local-regional recurrence or local-regional free survival; DM, incidence of distant metastasis; OS, overall survival, P, *p* value; n.s., not significant; TNT, total neoadjuvant treatment; n.r., not reported.

## Data Availability

The data are not publicly available.

## Supplementary Materials

The following are available online at https://www.mdpi.com/article/10.3390/cancers14153658/s1, Figure S1. (A) Discrimination ability of non-pCR for disease-free survival in the CAO/ARO/AIO-12 trial. (B) Discrimination ability of non-pCR for disease-free survival in Arm A and Arm B of the CAO/ARO/AIO-12 trial. Supplementary Methods: Time-dependent ROC curve analysis. Supplementary results of the time-dependent discrimination ability of pCR for DFS.

## References

[1] Kasi A., Abbasi S., Handa S., Al-Rajabi R., Saeed A., Baranda J., Sun W. (2020). Total Neoadjuvant Therapy vs Standard Therapy in Locally Advanced Rectal Cancer: A Systematic Review and Meta-analysis. JAMA Netw. Open.

[2] Petrelli F., Trevisan F., Cabiddu M., Sgroi G., Bruschieri L., Rausa E., Ghidini M., Turati L. (2020). Total Neoadjuvant Therapy in Rectal Cancer. Ann. Surg..

[3] Cercek A., Roxburgh C.S., Strombom P., Smith J.J., Temple L.K., Nash G.M., Guillem J.G., Paty P.B., Yaeger R., Stadler Z.K. (2018). Adoption of Total Neoadjuvant Therapy for Locally Advanced Rectal Cancer: A Systematic Review and Meta-analysis of Treatment Outcomes. JAMA Oncol..

[4] Fokas E., Glynne-Jones R., Appelt A., Beets-Tan R., Beets G., Haustermans K., Marijnen C., Minsky B.D., Ludmir E., Quirke P. (2020). Outcome measures in multimodal rectal cancer trials. Lancet Oncol..

[5] Rödel C., Graeven U., Fietkau R., Hohenberger W., Hothorn T., Arnold D., Hofheinz R.-D., Ghadimi M., Wolff H.A., Lang-Welzenbach M. (2015). Oxaliplatin added to fluorouracil-based preoperative chemoradiotherapy and postoperative chemotherapy of locally advanced rectal cancer (the German CAO/ARO/AIO-04 study): Final results of the multicentre, open-label, randomised, phase 3 trial. Lancet Oncol..

[6] Carvalho C., Glynne-Jones R. (2017). Challenges behind proving efficacy of adjuvant chemotherapy after preoperative chemoradiation for rectal cancer. Lancet Oncol..

[7] Breugom A.J., Swets M., Bosset J.-F., Collette L., Sainato A., Cionini L., Glynne-Jones R., Counsell N., Bastiaannet E., Broek C.B.M.V.D. (2015). Adjuvant chemotherapy after preoperative (chemo)radiotherapy and surgery for patients with rectal cancer: A systematic review and meta-analysis of individual patient data. Lancet Oncol..

[8] Voss R.K., Lin J.C., Roper M.T., Al-Temimi M.H., Ruan J.H., Tseng W.H., Tam M., Sherman M.J., Klaristenfeld D.D., Tomassi M.J. (2020). Adjuvant Chemotherapy Does Not Improve Recurrence-Free Survival in Patients With Stage 2 or Stage 3 Rectal Cancer After Neoadjuvant Chemoradiotherapy and Total Mesorectal Excision. Dis. Colon Rectum.

[9] Xu Z., Mohile S.G., Tejani M.A., Becerra A.Z., Probst C.P., Aquina C.T., Hensley B.J., Arsalanizadeh R., Noyes K., Monson J.R. (2017). Poor compliance with adjuvant chemotherapy use associated with poorer survival in patients with rectal cancer: An NCDB analysis. Cancer.

[10] Bahadoer R.R., A Dijkstra E., van Etten B., Marijnen C.A.M., Putter H., Kranenbarg E.M.-K., Roodvoets A.G.H., Nagtegaal I.D., Beets-Tan R.G.H., Blomqvist L.K. (2021). Short-course radiotherapy followed by chemotherapy before total mesorectal excision (TME) versus preoperative chemoradiotherapy, TME, and optional adjuvant chemotherapy in locally advanced rectal cancer (RAPIDO): A randomised, open-label, phase 3 trial. Lancet Oncol..

[11] Sclafani F., Corrò C., Koessler T. (2021). Debating Pros and Cons of Total Neoadjuvant Therapy in Rectal Cancer. Cancers.

[12] Conroy T., Bosset J.-F., Etienne P.-L., Rio E., François E., Mesgouez-Nebout N., Vendrely V., Artignan X., Bouché O., Gargot D. (2021). Neoadjuvant chemotherapy with FOLFIRINOX and preoperative chemoradiotherapy for patients with locally advanced rectal cancer (UNICANCER-PRODIGE 23): A multicentre, randomised, open-label, phase 3 trial. Lancet Oncol..

[13] Jin J., Tang Y., Hu C., Jiang L.-M., Jiang J., Li N., Liu W.-Y., Chen S.-L., Li S., Lu N.-N. (2022). Multicenter, Randomized, Phase III Trial of Short-Term Radiotherapy Plus Chemotherapy Versus Long-Term Chemoradiotherapy in Locally Advanced Rectal Cancer (STELLAR). J. Clin. Oncol..

[14] Garcia-Aguilar J., Patil S., Gollub M.J., Kim J.K., Yuval J.B., Thompson H.M., Verheij F.S., Omer D.M., Lee M., Dunne R.F. (2022). Organ Preservation in Patients with Rectal Adenocarcinoma Treated with Total Neoadjuvant Therapy. J. Clin. Oncol..

[15] George T.J., Allegra C.J., Yothers G. (2015). Neoadjuvant Rectal (NAR) Score: A New Surrogate Endpoint in Rectal Cancer Clinical Trials. Curr. Color. Cancer Rep..

[16] Fokas E., Ströbel P., Fietkau R., Ghadimi M., Liersch T., Grabenbauer G.G., Hartmann A., Kaufmann M., Sauer R., Graeven U. (2017). Tumor Regression Grading After Preoperative Chemoradiotherapy as a Prognostic Factor and Individual-Level Surrogate for Disease-Free Survival in Rectal Cancer. JNCI J. Natl. Cancer Inst..

[17] Fokas E., Fietkau R., Hartmann A., Hohenberger W., Grützmann R., Ghadimi M., Liersch T., Ströbel P., Grabenbauer G., Graeven U. (2018). Neoadjuvant rectal score as individual-level surrogate for disease-free survival in rectal cancer in the CAO/ARO/AIO-04 randomized phase III trial. Ann. Oncol..

[18] Fokas E., Schlenska-Lange A., Polat B., Klautke G., Grabenbauer G.G., Fietkau R., Kuhnt T., Staib L., Brunner T., Grosu A.-L. (2022). Chemoradiotherapy Plus Induction or Consolidation Chemotherapy as Total Neoadjuvant Therapy for Patients With Locally Advanced Rectal Cancer. JAMA Oncol..

[19] Fokas E., Allgäuer M., Polat B., Klautke G., Grabenbauer G.G., Fietkau R., Kuhnt T., Staib L., Brunner T., Grosu A.-L. (2019). Randomized Phase II Trial of Chemoradiotherapy Plus Induction or Consolidation Chemotherapy as Total Neoadjuvant Therapy for Locally Advanced Rectal Cancer: CAO/ARO/AIO-12. J. Clin. Oncol..

[20] Dworak O., Keilholz L., Hoffmann A. (1997). Pathological features of rectal cancer after preoperative radiochemotherapy. Int. J. Colorectal Dis..

[21] Valentini V., Van Stiphout R.G., Lammering G., Gambacorta M.A., Barba M.C., Bebenek M., Bonnetain F., Bosset J.-F., Bujko K., Cionini L. (2011). Nomograms for Predicting Local Recurrence, Distant Metastases, and Overall Survival for Patients With Locally Advanced Rectal Cancer on the Basis of European Randomized Clinical Trials. J. Clin. Oncol..

[22] Ballarini N.M., Thomas M., Rosenkranz G.K., Bornkamp B. (2021). subtee: An R Package for Subgroup Treatment Effect Estimation in Clinical Trials. J. Stat. Softw..

[23] Bujko K., Nowacki M.P., Nasierowska-Guttmejer A., Michalski W., Bebenek M., Kryj M. (2006). Long-term results of a randomized trial comparing preoperative short-course radiotherapy with preoperative conventionally fractionated chemoradiation for rectal cancer. Br. J. Surg..

[24] Ngan S.Y., Burmeister B., Fisher R.J., Solomon M., Goldstein D., Joseph D., Ackland S.P., Schache D., McClure B., McLachlan S.-A. (2012). Randomized Trial of Short-Course Radiotherapy Versus Long-Course Chemoradiation Comparing Rates of Local Recurrence in Patients with T3 Rectal Cancer: Trans-Tasman Radiation Oncology Group Trial 01.04. J. Clin. Oncol..

[25] Erlandsson J., Holm T., Pettersson D., Berglund A., Cedermark B., Radu C., Johansson H., Machado M., Hjern F., Hallböök O. (2017). Optimal fractionation of preoperative radiotherapy and timing to surgery for rectal cancer (Stockholm III): A multicentre, randomised, non-blinded, phase 3, non-inferiority trial. Lancet Oncol..

[26] Erlandsson J., Lörinc E., Ahlberg M., Pettersson D., Holm T., Glimelius B., Martling A. (2019). Tumour regression after radiotherapy for rectal cancer–Results from the randomised Stockholm III trial. Radiother. Oncol..

[27] Probst C.P., Becerra A.Z., Aquina C.T., Tejani M.A., Wexner S.D., Garcia-Aguilar J., Remzi F.H., Dietz D.W., Monson J.R., Fleming F.J. (2015). Extended Intervals after Neoadjuvant Therapy in Locally Advanced Rectal Cancer: The Key to Improved Tumor Response and Potential Organ Preservation. J. Am. Coll. Surg..

[28] Wolthuis A.M., Penninckx F., Haustermans K., De Hertogh G., Fieuws S., Van Cutsem E., D’Hoore A. (2012). Impact of Interval between Neoadjuvant Chemoradiotherapy and TME for Locally Advanced Rectal Cancer on Pathologic Response and Oncologic Outcome. Ann. Surg. Oncol..

[29] Petrelli F., Borgonovo K., Cabiddu M., Ghilardi M., Lonati V., Barni S. (2017). Pathologic complete response and disease-free survival are not surrogate endpoints for 5-year survival in rectal cancer: An analysis of 22 randomized trials. J. Gastrointest. Oncol..

[30] Rödel C., Liersch T., Becker H., Fietkau R., Hohenberger W., Hothorn T., Graeven U., Arnold D., Lang-Welzenbach M., Raab H.-R. (2012). Preoperative chemoradiotherapy and postoperative chemotherapy with fluorouracil and oxaliplatin versus fluorouracil alone in locally advanced rectal cancer: Initial results of the German CAO/ARO/AIO-04 randomised phase 3 trial. Lancet Oncol..

[31] Deng Y., Chi P., Lan P., Wang L., Chen W., Cui L., Chen D., Cao J., Wei H., Peng X. (2019). Neoadjuvant Modified FOLFOX6 With or Without Radiation Versus Fluorouracil Plus Radiation for Locally Advanced Rectal Cancer: Final Results of the Chinese FOWARC Trial. J. Clin. Oncol..

[32] Deng Y., Chi P., Lan P., Wang L., Chen W., Cui L., Chen D., Cao J., Wei H., Peng X. (2016). Modified FOLFOX6 With or Without Radiation Versus Fluorouracil and Leucovorin with Radiation in Neoadjuvant Treatment of Locally Advanced Rectal Cancer: Initial Results of the Chinese FOWARC Multicenter, Open-Label, Randomized Three-Arm Phase III Trial. J. Clin. Oncol..

[33] Jiao D., Zhang R., Gong Z., Liu F., Chen Y., Yu Q., Sun L., Duan H., Zhu S., Liu F. (2015). Fluorouracil-based preoperative chemoradiotherapy with or without oxaliplatin for stage II/III rectal cancer: A 3-year follow-up study. Chin. J. Cancer Res..

[34] Glynne-Jones R., Grainger J., Harrison M., Ostler P., Makris A. (2006). Neoadjuvant chemotherapy prior to preoperative chemoradiation or radiation in rectal cancer: Should we be more cautious?. Br. J. Cancer.

[35] Yothers G., George T.J., Allegra C.J., Bosset J.-F., Bujko K., Collette L., O’Connell M.J., Doyen J., Fernandez-Martos C., Seitz J.F. (2016). Predictive validity of NeoAdjuvant Rectal (NAR) Score and pathologic complete response (ypCR) for overall survival (OS) as surrogate endpoints in rectal cancer clinical trial. J. Clin. Oncol..

[36] Trakarnsanga A., Gönen M., Shia J., Nash G.M., Temple L.K., Guillem J.G., Paty P.B., Goodman K.A., Wu A., Gollub M. (2014). Comparison of Tumor Regression Grade Systems for Locally Advanced Rectal Cancer After Multimodality Treatment. JNCI: J. Natl. Cancer Inst..

[37] Kamarudin A.N., Cox T., Kolamunnage-Dona R. (2017). Time-dependent ROC curve analysis in medical research: Current methods and applications. BMC Med Res. Methodol..

[38] Blanche P. TimeROC: Time-Dependent ROC Curve and AUC for Censored Survival Data. R Package Version 02. https://cran.r-project.org/web/packages/timeROC/timeROC.pdf.

[39] Heagerty P.J., Saha-Chaudhuri P., Saha-Chaudhuri M.P. (2012). Package ‘risksetROC’.

