# Supplementary material for: Total Neoadjuvant Therapy for Rectal Cancer in the CAO/ARO/AIO-12 Randomized Phase 2 Trial: Early Surrogate Endpoints Revisited
Source: Cancers (Basel). 2022 Jul 27;14(15):3658. doi: 10.3390/cancers14153658 (PMC9367426; doi:10.3390/cancers14153658)
Supplement: Supplementary file 1 [file cancers-14-03658-s001.zip › cancers-1786248-supplementary.pdf]

cancers

Supplementary Material: Total Neoadjuvant Therapy for Rectal Cancer in the CAO/ARO/AIO-12 Randomized Phase 2 Trial: Early Surrogate Endpoints Revisited

Markus Diefenhardt *, Anke Schlenska-Lange, Thomas Kuhnt, Simon Kirste, Pompiliu Piso, Wolf O. Bechstein, Guido Hildebrandt, Michael Ghadimi, Ralf-Dieter Hofheinz, Claus Rödel, Emmanouil Fokas on behalf of the German Rectal Cancer Study Group

Supplementary Methods

Time-Dependent ROC Curve Analysis

Time-varying discrimination accuracy of non-pCR for DFS was analyzed in the complete cohort with the risksetROC (Incident sensitivity and dynamic specificity estimator) package and independently for Arm A and Arm B with the timeroc (Inverse Probability of Censoring Weighting estimator) package [37, 38, 39].

Supplementary References

37. Kamarudin AN, Cox T, Kolamunnage-Dona R. Time-dependent ROC curve analysis in medical research: current methods and applications. BMC Med Res Methodol. 2017;17(1):53.
38. Blanche P. TimeROC: Time-dependent ROC curve and AUC for censored survival data. R package version 02, URL https://cran.r-project.org/web/ packages/timeROC/timeROC.pdf.
39. Heagerty PJ, Saha-Chaudhuri P, Saha-Chaudhuri MP. Package 'risksetROC'. 2012

Supplementary Results

The discrimination ability of non-pCR for DFS in the complete cohort was visually assessed over time (C-Index 0.58, Figure S1A). At 3-year, non-pCR achieved a discrimination ability AUC of 0.61 in Arm A, and AUC of 0.55 in Arm B for DFS. Discrimination ability after 36 months was not significantly different between treatment arms (DeLong's test P=0.13), whereas discrimination ability of non-pCR for DFS remained constantly weaker in Arm B over time and seems to get worse with longer follow up period (Figure S1B).

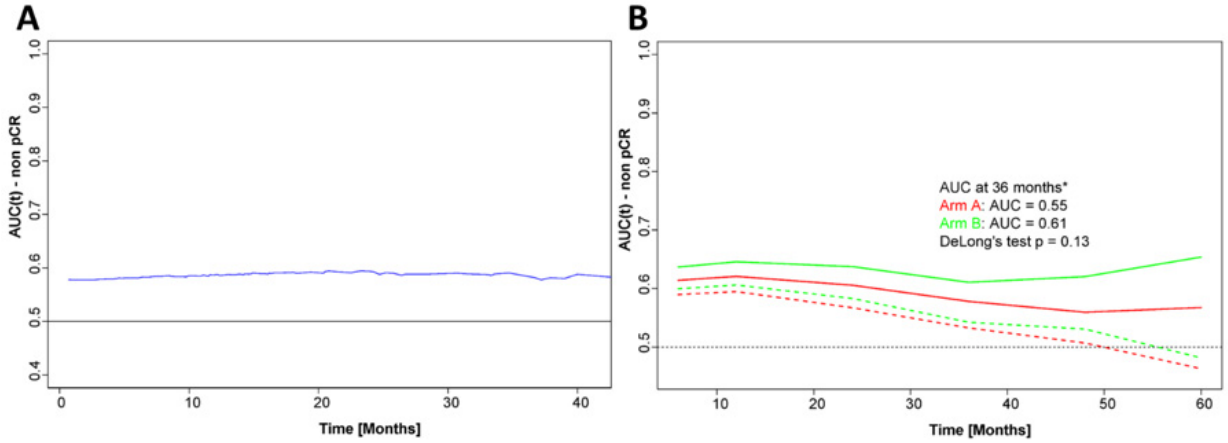

Figure S1. (A) Discrimination ability of non-pCR for disease-free survival in the CAO/ARO/AIO-12 trial. **(B)** Discrimination ability of non-pCR for disease-free survival in Arm A and Arm B of the CAO/ARO/AIO-12 trial. Upper border of the 95% confidence interval plotted as dashed line. AUC curve over time calculated and plotted with "risksetROC" (**A**) and "timeroc" (**B**) package in R.*all events after 36months were censored
